# Exploring the mediating role of physical activity levels in the relationship between physical literacy and physical fitness in Chinese university students

**DOI:** 10.7717/peerj.20689

**Published:** 2026-02-03

**Authors:** Yingshuang Sun, Donglin Hu

**Affiliations:** 1 Department of Physical Education, Suzhou Institute of Industrial Technology, Suzhou, China; 2 College of Sports Industry and Leisure, Nanjing Sport Institute, Nanjing, Jiangsu Province, China; 3 Department of Physical Education, Nanjing Agricultural University, Nanjing, Jiangsu Province, China; 4 School of Physical Education and Educational Science, Tianjin University of Sport, Tianjin, China

**Keywords:** Physical activity, Physical fitness, Mediation, University students, Physical literacy

## Abstract

**Objective:**

This study investigated the relationships between physical literacy (PL), physical activity levels (PALs), and physical fitness among Chinese university students, with a particular focus on the mediating role of PALs and the consistency of findings across various analytical methods.

**Methods:**

Participants included 115 first-year students (79 males, 36 females) from Nanjing, China. PL was assessed using the Perceived Physical Literacy Instrument (PPLI-SC), PALs were measured using the Physical Activity Questionnaire for Adolescents (PAQ-A), and physical fitness was assessed through four indicators: body mass index (BMI), 50-m sprint, sit-and-reach, and 800/1,000-m run. Descriptive statistics, correlation, and regression analyses were conducted in SPSS 26.0. Mediation was tested using the PROCESS macro (Model 4, 5,000 bootstrap samples), and structural equation modeling (SEM) was applied using AMOS 26.0 for validation.

**Results:**

Males outperformed females in the 50-m sprint, while females exhibited superior flexibility and cardiorespiratory endurance; BMI was significantly higher in males (*p* < 0.01). PL was positively correlated with PALs (*r* = 0.584, *p* < 0.01), and both were significantly associated with all fitness indicators except BMI. Mediation analyses indicated that PALs fully mediated the associations between PL and sprint performance, flexibility, and cardiorespiratory endurance. For BMI, the PROCESS macro suggested a weak indirect effect, whereas SEM results were nonsignificant, highlighting the sensitivity of the results to the analytical method used.

**Conclusion:**

PL is strongly linked to physical fitness in Chinese university students, with PALs acting as a critical mediator. The inconsistent BMI findings underscore its complex determinants, which extend beyond physical activity. Promoting PL and PALs through university physical education programs may improve fitness outcomes. Future research should validate these findings using larger samples, longitudinal designs, and broader body composition indicators (*e.g.*, body fat percentage, muscle mass).

## Introduction

In recent years, insufficient physical activity among children and adolescents has emerged as a major global public health challenge (Bull et al., 2020). According to data from Guthold et al. (2020), based on World Health Organization (WHO)-coordinated global surveillance of adolescents aged 11–17, about 81% were insufficiently physically active in 2016, meaning that only around 19% met the recommended levels. In China, the compliance rate is approximately 34% (Liu et al., 2023), which is slightly higher than the global average but still low, indicating that more than two-thirds of Chinese adolescents fail to reach the recommended standards. This situation not only negatively affects physical health but is also closely associated with declines in mental health and physical fitness performance.

Against this backdrop, the concept of physical literacy (PL) has increasingly gained attention in both academia and educational practice. PL extends beyond physical skills and fitness to encompass motivation, confidence, knowledge, and understanding, and is regarded as a key determinant for promoting lifelong participation in physical activity (Whitehead, 2013). Previous studies have shown that PL is significantly associated with physical activity levels (PALs) and physical fitness in children and adolescents (Cairney et al., 2019; Jiang et al., 2025; Mayordomo-Pinilla et al., 2025). However, most of this research has focused on younger populations, while university freshmen—who are in a transitional stage characterized by changes in academic demands, lifestyle, and exercise habits—have received relatively little attention. As a result, the relationships among PL, PALs, and physical fitness in this unique age group remain insufficiently understood.

According to Whitehead (2013), physical literacy encompasses motivation, confidence, physical competence, and knowledge and understanding, which collectively underpin an individual’s capacity and willingness to engage in physical activity across the lifespan. Building on this foundation, Cairney et al. (2019) proposed an evidence-informed conceptual model positioning physical literacy as a determinant of health, operating partly through behavioral pathways such as participation in physical activity. Together, these perspectives suggest that physical literacy may influence physical fitness indirectly by shaping individuals’ engagement in physical activity. Although these theoretical propositions do not specify a formal mediation model, they provide a conceptual basis for examining physical activity levels as a potential pathway linking physical literacy to fitness outcomes. Given that physical activity represents the most direct behavioral expression of physical literacy, PALs serve as a theoretically grounded mediator linking literacy-related attributes to fitness outcomes. This rationale aligns with the behavioral pathway emphasized in the physical literacy–health model proposed by Cairney et al. (2019).

Physical fitness indicators have long been recognized as essential measures of health status in adolescents and young adults (Ortega et al., 2008). In China, these standardized indicators constitute core components of both university-level and national student physical fitness assessment systems, providing strong ecological validity for examining how PL and PALs translate into actual performance outcomes. A large body of research consistently shows that PALs are strongly linked to fitness outcomes, including explosive power, flexibility, and cardiorespiratory endurance (Lee et al., 2021; Singh, Pattisapu & Emery, 2020; Wu et al., 2023). However, the associations between PL, PALs, and body mass index (BMI) remain controversial (Cornish et al., 2020). Some studies have reported significant correlations between PL and BMI (Domínguez-Martín, Tárraga-López & López-Gil, 2024; Holler et al., 2019), whereas others have yielded inconsistent results (Anderson et al., 2019; Yan et al., 2024). This suggests that BMI may be influenced by multiple behavioral and biological factors. Therefore, clarifying how PL and PALs relate to BMI in university students is an important and unresolved question.

In light of this background, the present study focused on Chinese university freshmen to systematically examine the relationships among PL, PALs, and physical fitness indicators, with particular emphasis on the mediating role of PALs. To ensure robustness, the study employed multiple methods: traditional correlation and regression analyses, mediation analysis using the PROCESS macro, and structural equation modeling (SEM). This multi-method approach allows us to compare the consistency of mediation effects across different analytical frameworks, which has rarely been examined in previous literature. By integrating these empirical approaches, the study aimed to deepen understanding of the PL → PALs → fitness pathway and provide both theoretical and practical evidence for optimizing university physical education curricula and promoting student health. Overall, this study contributes to the existing literature by addressing an understudied population, clarifying an unresolved debate regarding BMI, and comparing the consistency of mediation effects across different analytical approaches.

## Methods

### Participants

A total of 115 first-year university students (79 males, 36 females) from a university in Nanjing, China, were recruited using cluster sampling between October 2024 and January 2025. All participants provided written informed consent prior to the study, and the protocol was approved by the Ethics Committee of Tianjin University of Sport (approval number: 2023011). Eligible participants were first-year students aged 18–22 with no physical disabilities or conditions that could hinder physical activity. Students with cardiovascular or respiratory diseases or those undergoing physical rehabilitation were excluded. Cluster sampling was used to randomly select classes, and all students in the chosen classes who met the eligibility criteria were invited to participate.

### Measures

#### Physical Literacy (PL)

PL was assessed using the Simplified Chinese version of the Perceived Physical Literacy Instrument (PPLI-SC), which includes nine items across three dimensions: motivation, confidence, and knowledge/understanding (Ma et al., 2020). Responses were rated on a 5-point Likert scale, with higher scores indicating greater levels of PL.

#### Physical Activity Levels (PALs)

PALs were measured using the Chinese version of the Physical Activity Questionnaire for Adolescents (PAQ-A). This instrument includes seven items assessing the frequency and intensity of physical activity over the previous seven days. The average score of the items was used as the PALs score, ranging from 1 to 5, with higher values indicating greater activity levels (Fühner et al., 2021). In the present study, the internal consistency reliability of the PAQ-A was good (Cronbach’s α = 0.83). The PAQ-A has been widely applied in samples aged 14–19 years, and our participants (mean age = 18.09 years) fall within this developmental range, supporting the appropriateness of using this instrument among first-year university students. Moreover, given the modest sample size (*n* = 115), confirmatory factor analysis (CFA) was not conducted, as CFA typically requires substantially larger samples to yield stable and reliable parameter estimates.

#### Physical fitness indicators

**Body Mass Index (BMI):** Calculated from measured height (m) and weight (kg) as weight/height^2^.

**Explosive Power (EP):** Assessed using the 50-m sprint time (s), with shorter times reflecting greater explosive power.

**Flexibility (F):** Evaluated using the sit-and-reach test (cm), with higher values indicating better flexibility.

**Cardiorespiratory Endurance (CE):** Assessed using a 1,000-m run for males and an 800-m run for females, with shorter times indicating better endurance.

### Data collection procedure

All tests were administered during physical education classes under the supervision of trained instructors. Height and weight were measured using standardized equipment, and physical fitness assessments were conducted following the National Student Physical Fitness Standard (Education Ministry of the People’s Republic of China, 2014). All fitness tests were conducted within a single session in the following order: 5-minute standardized warm-up, 50-m sprint, sit-and-reach test, and 800/1,000-m run. This protocol ensured consistency across participants and minimized procedural variability. Questionnaires were administered in a group setting, with researchers present to provide instructions and ensure accurate completion.

### Data analysis

#### Descriptive statistics and gender differences

All analyses were performed using IBM SPSS Statistics 26.0. Continuous variables were expressed as mean ± standard deviation, and categorical variables as frequencies and percentages. Independent-samples *t* tests were used to assess gender differences; when normality assumptions were not met, Mann–Whitney U tests were applied.

#### Correlation analysis

Pearson’s correlation coefficients were computed to examine the bivariate relationships between PL, PALs, and the four physical fitness indicators.

#### Regression analysis

Hierarchical regression analyses were conducted. First, the predictive effect of PL on PALs was examined. PALs was then added to the models to test its role in the relationships between PL and physical fitness indicators. Multicollinearity diagnostics indicated that all predictors had acceptable VIF values (all VIFs < 2.0), suggesting no multicollinearity concerns.

#### Mediation analysis (PROCESS Macro)

Mediation analyses were conducted using the PROCESS macro for SPSS (Model 4, 5000 bootstrap samples), with PALs as the mediator, PL as the independent variable, and physical fitness indicators as dependent variables. Mediation was considered significant if the 95% bootstrap confidence interval excluded zero.

#### Structural Equation Modeling (SEM)

To further validate the mediation effects, a simplified path model was specified in AMOS 26.0. Given the relatively small sample size (*n* = 115), total scores for PL and PALs were modeled as observed variables, and the four physical fitness indicators were included as outcome variables. Parameters were estimated using maximum likelihood estimation (MLE). Model fit was evaluated using *χ*^2^/df, Comparative Fit Index (CFI), Tucker–Lewis Index (TLI), Goodness-of-Fit Index (GFI), Adjusted Goodness-of-Fit Index (AGFI), and the Root Mean Square Error of Approximation (RMSEA). Indirect effects were tested using bootstrapping (5,000 resamples). In this study, SEM was applied primarily as a supplementary approach to cross-validate the mediation patterns observed in PROCESS, rather than to estimate a complex structural model, which minimizes concerns related to the relatively small sample size.

## Results

### Characteristics of participants

A total of 115 first-year university students were participated in this study (79 males and 36 females), with a mean age of 18.09 ± 0.74 years. The majority of participants were within the normal weight range (72.2%), while 9.6% were underweight, 11.3% were overweight, and 7.0% were obese. Gender comparisons indicated that males had significantly higher height, weight, and BMI than females (*p* < 0.01) (see Table 1).

### Descriptive statistics

Overall, participants reported a mean PL score of 19.86 ± 4.28 and a mean PALs score of 3.73 ± 0.62. Regarding physical fitness indicators, the average 50-m sprint time was 7.67 ± 0.51 s, the mean sit-and-reach performance was 12.10 ± 4.71 cm, and the average 800/1,000 m run time was 226.25 ± 24.75 s (see Table 2).

### Gender differences

Independent-samples *t*-tests revealed the following:

Males performed significantly better than females in the EP (*p* < 0.01).

Females exhibited significantly better performance in F (*p* < 0.01) and CE (*p* < 0.01).

Males had significantly higher BMI levels than females (*p* < 0.01).

No significant gender differences were observed in PL or PALs (*p* > 0.05).

### Correlation analysis

Pearson correlation analysis revealed the following:

PL was positively correlated with PALs (*r* = 0.584, *p* < 0.01).

PL was negatively correlated with EP and CE, and positively correlated with flexibility (*p* < 0.01), but not significantly related to BMI.

**Table 1:** Characteristics of participants (*n* = 115).

Variable	Total sample	Male (*n* = 79)	Female (*n* = 36)	*p*-value
Age (years)	18.09 ± 0.74	18.15 ± 0.79	17.94 ± 0.67	0.15
Height (m)	1.72 ± 0.08	1.76 ± 0.05	1.63 ± 0.05	<0.01
Weight (kg)	64.3 ± 14.0	69.03 ± 14.03	53.84 ± 5.80	<0.01
BMI	21.6 ± 3.62	22.20 ± 4.03	20.23 ± 1.89	<0.01
Weight category (n, %)	Normal: 83 (72.2%);	–	–	–
	Underweight: 11(9.6%);			
	Overweight: 13 (11.3%);			
	Obese: 8 (7.0%)			

**Notes.**

Note: Data are presented as mean ± SD unless otherwise stated.

BMI Body Mass Index

**Table 2:** Descriptive statistics of PL, PALs, and fitness indicators (*n* = 115).

Variable	Total	Male (*n* = 79)	Female (*n* = 36)	*t*-value	*p*-value
PL	19.86 ± 4.28	19.81 ± 4.62	19.97 ± 3.47	−0.187	>0.05
PALs	3.73 ± 0.62	3.69 ± 0.67	3.81 ± 0.47	−1.060	>0.05
EP	7.67 ± 0.51	7.56 ± 0.52	7.93 ± 0.37	−3.899	<0.01
F	12.10 ± 4.71	11.21 ± 4.70	14.06 ± 4.18	−3.114	<0.01
CE	226.25 ± 24.75	231.94 ± 23.50	213.78 ± 23.06	3.865	<0.01

**Notes.**

Note PL Physical Literacy PALs Physical Activity Levels EP Explosive Power (50 m-sprint, sec) F Flexibility (sit-and-reach, cm) CE Cardiorespiratory Endurance (800/1,000 m run, sec)

PALs were negatively correlated with EP and CE, and positively correlated with F (*p* < 0.01), while their correlation with BMI was weak and non-significant.

Overall, both PL and PALs were significantly associated with most physical fitness indicators, except for BMI (see Table 3).

### Regression analysis

The results of the hierarchical regression analysis showed:

PL significantly predicted PALs in a positive direction (*β* = 0.584, *p* < 0.01), explaining 34.1% of the variance (see Table 4).

With respect to the physical fitness indicators:

In the models of EP, CE, and F, after PALs were introduced, the predictive effect of PL was no longer significant, while PALs showed significant predictive effects (*p* < 0.01), indicating full mediation.

In the BMI model, the direct effect of PL on BMI was not significant, but PALs significantly predicted BMI (*p* < 0.05), suggesting that PALs may play a role in the relationship between PL and BMI, although the effect was relatively weak (see Table 5).

**Table 3:** Pearson correlations between PL, PALs, and physical fitness indicators (*n* = 115).

Variable	PL	PALs	BMI	EP	CE	F
PL	1	0.584^**^	0.053	−0.395^**^	−0.332^**^	0.278^**^
PALs		1	−0.148	−0.664^**^	−0.471^**^	0.500^**^
BMI			1	0.127	0.390^**^	−0.214^*^
EP				1	0.316^**^	−0.283^**^
CE					1	−0.322^**^
F						1

**Notes.**

* *p*  <  0.05.

** *p*  <  0.01.

PL Physical Literacy PALs Physical Activity Levels EP Explosive Power (50-m sprint) F Flexibility(sit-and-reach) CE Cardiorespiratory Endurance (800/1,000 m run) BMI Body Mass Index

**Table 4:** Regression analysis of physical literacy predicting physical activity levels (*n* = 115).

Independent variable	B	SE	*β*	t	*p*	R^2^	F
PL	0.084	0.011	0.584	7.648	<0.01	0.341	58.493^**^

**Notes.**

** *p* < 0.01.

PL Physical Literacy PALs Physical Activity Levels

**Table 5:** Hierarchical regression analyses of physical literacy and physical activity levels predicting fitness indicators (*n* = 115).

Dependent variable	Model	Independent variable	B	SE	*β*	*t*	*p*	R^2^	ΔR^2^
BMI	1	PL	0.045	0.079	0.053	0.561	>0.05	0.003	–
	2	PL	0.179	0.096	0.212	1.866	>0.05	0.052	0.049^*^
		PALs	−1.596	0.665	−0.272	−2.399	<0.05		
EP	1	PL	−0.047	0.010	−0.395	−4.572	<0.01	0.156	–
	2	PL	−0.001	0.010	−0.011	−0.131	>0.05	0.440	0.284^**^
		PALs	−0.539	0.071	−0.657	−7.544	<0.01		
CE	1	PL	−1.919	0.514	−0.332	−3.737	<0.01	0.110	–
	2	PL	−0.496	0.592	−0.086	−0.837	>0.05	0.227	0.117^**^
		PALs	−16.923	4.113	−0.421	−4.115	<0.01		
F	1	PL	0.307	0.100	0.278	3.078	<0.01	0.077	–
	2	PL	−0.023	0.111	−0.021	−0.208	>0.05	0.250	0.173^**^
		PALs	3.922	0.772	0.512	5.081	<0.01		

**Notes.**

* *p* < 0.05.

** *p* < 0.01.

PL Physical Literacy PALs Physical Activity Levels EP Explosive Power (50-m sprint) F Flexibility (sit-and-reach) CE Cardiorespiratory Endurance (800/1,000 m run) BMI Body Mass Index

### Mediation analysis (PROCESS)

The PROCESS macro (Model 4, Bootstrap = 5,000) was used to further examine the mediating role of PALs:

In the models of EP, CE, and F, the indirect effects of PALs were all significant (95% CI excluded zero), while the direct effects were nonsignificant, indicating that PALs fully mediated the relationships between PL and these fitness indicators.

In the BMI model, the indirect effect of PALs was statistically significant (Effect = −0.134, 95% CI [−0.270, −0.026]); but the effect size was small. This suggests that PALs may mediate the relationship between PL and BMI to some extent, although its explanatory power is weaker compared to other fitness indicators (see Table 6).

**Table 6:** Mediation analysis using PROCESS macro (Bootstrap = 5,000, *n* = 115).

Dependent variable	Path	Effect	SE	*t*	*p*	95% CI ** Lower**	**95% CI** ** Upper**	**Conclusion**
BMI	PL → PALs	0.084	0.011	7.648	<0.01	0.062	0.106	Significant
	PL → BMI (Direct effect)	0.179	0.096	1.866	>0.05	−0.011	0.369	Nonsignificant
	PALs → BMI	−1.596	0.665	−2.399	<0.05	−2.915	−0.278	Significant
	**Indirect effect**	−0.134	0.063	–	–	−0.270	−0.026	Significant
EP	PL → PALs	0.084	0.011	7.648	<0.01	0.062	0.106	Significant
	PL → EP (Direct effect)	−0.001	0.010	−0.131	>0.05	−0.022	0.019	Nonsignificant
	PALs → EP	−0.539	0.072	−7.544	<0.01	−0.681	−0.398	Significant
	**Indirect effect**	−0.045	0.008	–	–	−0.061	−0.030	Significant
CE	PL → PALs	0.084	0.011	7.648	<0.01	0.062	0.106	Significant
	PL → CE (Direct effect)	−0.496	0.592	−0.837	>0.05	−1.670	0.678	Nonsignificant
	PALs → CE	−16.923	4.113	−4.115	<0.01	−25.072	−8.774	Significant
	**Indirect effect**	−1.424	0.433	–	–	−2.328	−0.642	Significant
F	PL → PALs	0.084	0.011	7.648	<0.01	0.062	0.106	Significant
	PL → F (Direct effect)	−0.023	0.111	−0.209	>0.05	−0.243	0.197	Nonsignificant
	PALs → F	3.922	0.772	5.081	<0.01	2.392	5.451	Significant
	**Indirect effect**	0.330	0.078	–	–	0.185	0.494	Significant

**Notes.**

PL Physical Literacy PALs Physical Activity Levels BMI Body Mass Index EP Explosive Power (50-m sprint) CE Cardiorespiratory Endurance (800/1,000 m run) F Flexibility (sit-and-reach)

### Structural Equation Modeling (SEM) analysis

To further validate the mediation effect, a path model (PL → PALs → Physical Fitness) was constructed using AMOS 26.0. Given the limited sample size, the total scores of PL and PALs were treated as observed variables, while physical fitness indicators (BMI, EP, F, and CE) were included as outcome variables.

#### Model fit

The overall model fit was acceptable: *χ*^2^/*df* = 2.729 (<3), GFI = 0.932, CFI = 0.909, IFI = 0.911, AGFI = 0.857. Although RMSEA = 0.123 (90% CI [0.069–0.180]) was slightly higher than the ideal threshold, it remains within an acceptable range, considering the small sample size.

#### Path coefficients

The path analysis results indicated the following:

PL significantly predicted PALs positively (Estimate = 0.084, *p* < 0.001);

PALs significantly predicted EP, F, and CE (all *p* < 0.001);

The path from PALs to BMI was nonsignificant (Estimate = −0.871, *p* = 0.132).

#### Mediation effect verification

The bootstrap results from SEM showed that the indirect effects of PALs between PL and EP, F, and CE were all significant (95% CI excluded zero). In the BMI model, however, the indirect effect was nonsignificant (95% CI included zero). This inconsistency may arise from methodological differences: while PROCESS detected a significant effect when analyzing BMI as a single outcome, the effect weakened when BMI was modeled simultaneously with other outcomes in SEM, suggesting potential instability. Previous studies have also reported that among university students with relatively homogeneous weight status, the relationship between BMI and physical activity tends to be weaker and more sensitive to limited statistical power (Jacob et al., 2021; Lee et al., 2021; Robbins et al., 2012).

**Figure 1:**
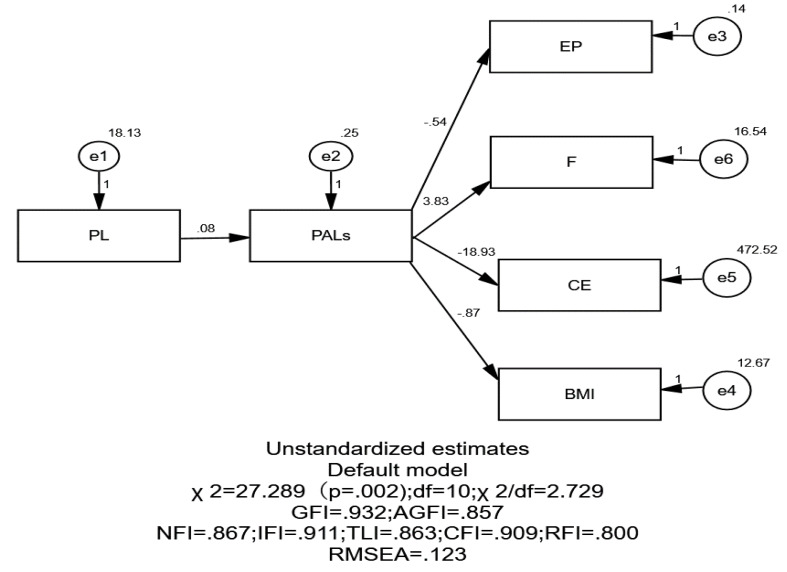
Structural equation modeling of physical literacy, physical activity levels and fitness indicators. The diagram presents the unstandardized path coefficients estimated in the SEM analysis. PL predicts PALs, which in turn predict four physical fitness indicators: explosive power (EP), flexibility (F), cardiorespiratory endurance (CE), and body mass index (BMI). Error terms (e1–e6) and their variances are shown for each observed variable. Model fit indices (*χ*^2^, df, GFI, AGFI, NFI, IFI, TLI, CFI, RFI, RMSEA) are reported beneath the diagram.

Overall, the findings consistently support that PALs play a crucial mediating role between PL and major fitness indicators (EP, F, and CE), whereas the mediation effect on BMI is relatively weak and warrants further examination in studies with larger sample sizes or multi-indicator designs (see Table 7 and Fig. 1).

## Discussion

### Main findings

This study systematically examined the associations between PL, PALs, and physical fitness in a sample of Chinese university freshmen. Three key findings emerged. First, PL was significantly positively correlated with PALs, and PL significantly predicted PALs, supporting the theoretical proposition that literacy facilitates behavior. Second, PALs played a central mediating role in the relationships between PL and EP, F, and CE. In these models, PALs demonstrated full mediation, indicating that PL exerts its influence on physical fitness primarily through increased activity levels. Third, in the BMI model, PROCESS analysis suggested a small but significant mediation effect of PALs, while SEM revealed a nonsignificant effect, highlighting the limited and unstable role of PALs in the PL–BMI relationship.

**Table 7:** Path coefficients and mediation effects from SEM (*n* = 115).

Path	Estimate	S.E.	C.R.	*p*-value	Result
PL → PALs	0.084	0.011	7.682	^***^	Significant
PALs → EP	−0.545	0.058	−9.471	^***^	Significant
PALs → F	3.828	0.621	6.163	^***^	Significant
PALs → CE	−18.933	3.319	−5.704	^***^	Significant
PALs → BMI	−0.871	0.544	−1.602	0.109	Nonsignificant

**Notes.**

**Note:** *** *p* < 0.001.

PL Physical Literacy PALs Physical Activity Levels EP Explosive Power (50-m sprint) F Flexibility (sit-and-reach) CE Cardiorespiratory Endurance (800/1,000 m run) BMI Body Mass Index

### Comparison with previous studies

Previous studies have consistently reported a positive association between PL and PALs among adolescents and young adults (Angulo et al., 2020; Choi et al., 2018), which aligns with the present findings. Furthermore, the link between PL and physical fitness has also been observed in different populations. For example, Yan et al. (2022) reported significant associations of PL with endurance and flexibility in Chinese young adults.

Regarding mediation effects, international evidence suggests that PALs may act as a mediator between PL and health or fitness outcomes (Caldwell et al., 2020). Although similar associations have been documented in younger age groups, research on university freshmen remains limited. The present study extends these findings to Chinese university freshmen, providing additional empirical support for the mediating role of PALs.

The inconsistent BMI findings align with prior evidence indicating that associations between physical activity and adiposity-related indices in youth and young adults are generally small and sensitive to measurement and confounding factors (Dalene et al., 2017; Marques et al., 2016). In a sample of young adults, Yan et al. (2024) also observed a statistically non-significant PA–BMI association, suggesting that BMI may be less responsive to behavioral variance than performance-based fitness outcomes. This helps explain why in our study, PROCESS detected a significant mediation effect of PALs on BMI, whereas SEM did not: the small and unstable effect may be more easily captured in single-outcome regression with resampling, but attenuated under the more conservative global modeling constraints of SEM.

### Possible mechanisms

First, higher PL enhances motivation, confidence, and skill acquisition, which in turn promotes PALs. Increased PALs subsequently improve CE, EP, and F through more frequent aerobic and anaerobic exercises. This mechanism is consistent with the theoretical framework that links PL, PALs, and physical fitness.

Second, although physical activity contributes to energy balance, BMI is simultaneously influenced by several other factors, including dietary habits, basal metabolic rate, and genetics (Hall & Guo, 2017). Consequently, in short-term or small-sample studies, PALs may show unstable or nonsignificant effects on BMI. This complexity supports the interpretation that BMI-related pathways are less consistent than performance-based fitness outcomes.

### Methodological considerations and limitations

This study employed both PROCESS and SEM to test mediation, enhancing the robustness of the conclusions. However, differences between the two methods were observed in the BMI model. The PROCESS macro, which analyzes outcomes individually and uses resampling to assess mediation, may detect effects more readily, whereas SEM estimates all parameters simultaneously and adopts a more conservative approach, which can attenuate weaker effects (Hayes, 2017). Such methodological discrepancies are not uncommon in mediation research (Hayes & Scharkow, 2013). Furthermore, by applying both PROCESS and SEM, the present study provides methodological insight into how weak mediation effects may behave differently across analytical approaches.

Several limitations should be acknowledged. The relatively small sample size (*n* = 115) may limit the stability of SEM results. Additionally, the discrepancy observed between PROCESS and SEM in the BMI pathway should be interpreted cautiously, as the small sample size may have reduced the statistical power of SEM, leading to attenuated estimates for weaker effects. Furthermore, the sample was drawn from a single university in Nanjing, which limits the generalizability of the findings to broader populations. BMI, as a single indicator, does not fully capture body composition (*e.g.*, fat mass, muscle mass). Finally, the cross-sectional design precludes causal inferences. Future studies should adopt longitudinal or experimental designs to more rigorously examine the causal relationships among PL, PALs, and physical fitness.

### Practical implications and future directions

From a practical perspective, the findings underscore the importance of enhancing PL to promote PALs, thereby improve physical fitness among university students. University physical education programs should prioritize developing PL through curricular and extracurricular activities, aiming to foster motivation, competence, and confidence.

Future studies should:

• use larger and more diverse samples across different regions and grade levels;
• incorporate more comprehensive measures of body composition (*e.g.*, body fat percentage, muscle mass) beyond BMI;
• employ longitudinal or experimental designs to establish causal pathways;
• examine psychological factors such as motivation and self-efficacy as potential moderators of the PL–PALs relationship.

## Conclusion

Drawing on data from 115 Chinese university freshmen, this study demonstrated that PL is significantly associated with PALs and that PALs fully mediate the relationships between PL and key fitness outcomes, including EP, F, and CE. While the mediation effect of PALs on BMI was significant in PROCESS but nonsignificant in SEM, this discrepancy likely reflects methodological differences and the complex determinants of BMI.

Overall, the findings confirm the pathway *“PL*→* PALs*→* fitness”*, emphasizing PALs as a behavioral bridge that links physical literacy to performance. This study extends existing evidence to the underrepresented population of Chinese university freshmen and underscores the importance of promoting PL within higher education. Practically, the results suggest that enhancing PL through university physical education programs and interventions may increase PALs, thereby improving fitness outcomes.

Future research should validate these findings with larger and more diverse samples, adopt longitudinal designs, and incorporate broader body composition indicators (*e.g.*, body fat percentage, muscle mass). Additionally, examining psychosocial moderators such as motivation and self-efficacy will provide deeper insight into how PL translates into sustained active lifestyles.

##  Supplemental Information

10.7717/peerj.20689/supp-1 Supplemental Information 1 STROBE Checklist

10.7717/peerj.20689/supp-2 Supplemental Information 2 Participant demographics, physical literacy, physical activity levels, and various physical fitness indicators, including BMI, explosive power, cardiorespiratory endurance, flexibility, and self-reported physical activity level

10.7717/peerj.20689/supp-3 Supplemental Information 3 Codebook for Dataset Variables: Definitions and Categories for Participant Demographics, Physical Activity, and Fitness Indicators

## Additional Information and Declarations

### Data Availability

The following information was supplied regarding data availability: The data is available in the Supplemental File.
