# Supplementary material for: Exploring the mediating role of physical activity levels in the relationship between physical literacy and physical fitness in Chinese university students
Source: PeerJ. 2026 Feb 3;14:e20689. doi: 10.7717/peerj.20689 (PMC12880097; doi:10.7717/peerj.20689)
Supplement: Supplemental Information 1 [file peerj-14-20689-s001.docx]

STROBE Statement—checklist of items that should be included in reports of observational studies

|  | Item No. | Recommendation | Page  No. | Relevant text from manuscript |
| --- | --- | --- | --- | --- |
| **Title and abstract** | 1 | (*a*) Indicate the study's design with a commonly used term in the title or the abstract | 1 | The study design is clearly indicated in the title: "Exploring the mediating role of physical activity levels in the relationship between physical literacy and physical fitness in Chinese university students. |
|  |  | (*b*) Provide in the abstract an informative and balanced summary of what was done and what was found | 1 | The abstract provides a concise summary of the study's purpose, methods, results, and conclusion, indicating that physical literacy (PL) is strongly linked to physical fitness and that physical activity levels (PALs) mediate the relationship between PL and fitness outcomes. |
| Introduction | | | |  |
| Background/rationale | 2 | Explain the scientific background and rationale for the investigation being reported | 2 | The introduction provides the scientific background and rationale for investigating the relationship between physical literacy (PL) and physical fitness, as well as the mediating role of physical activity levels (PALs). |
| Objectives | 3 | State specific objectives, including any prespecified hypotheses | 3 | The study aims to explore the relationship between PL, PALs, and physical fitness, with a focus on PALs as a mediator. |
| Methods | | | |  |
| Study design | 4 | Present key elements of study design early in the paper | 3 | The study is described as a cross-sectional study examining relationships between PL, PALs, and physical fitness among Chinese university students. |
| Setting | 5 | Describe the setting, locations, and relevant dates, including periods of recruitment, exposure, follow-up, and data collection | 3 | The research was conducted at a university in Nanjing, China, between October 2024 and January 2025. |
| Participants | 6 | (*a*) *Cohort study*—Give the eligibility criteria, and the sources and methods of selection of participants. Describe methods of follow-up  *Case-control study*—Give the eligibility criteria, and the sources and methods of case ascertainment and control selection. Give the rationale for the choice of cases and controls  *Cross-sectional study*—Give the eligibility criteria, and the sources and methods of selection of participants | 3 | **Eligibility Criteria:** First-year students aged 18-22 with no physical disabilities or conditions hindering physical activity. Students with cardiovascular or respiratory diseases or undergoing rehabilitation were excluded.  **Sources and Methods of Selection:** Cluster sampling was used to randomly select classes from a university in Nanjing, China. All eligible students in the selected classes were invited to participate. |
|  |  | (*b*) *Cohort study*—For matched studies, give matching criteria and number of exposed and unexposed  *Case-control study*—For matched studies, give matching criteria and the number of controls per case | 3 |  |
| Variables | 7 | Clearly define all outcomes, exposures, predictors, potential confounders, and effect modifiers. Give diagnostic criteria, if applicable | 4 | The key variables—PL, PALs, and physical fitness indicators (BMI, 50-m sprint, flexibility, 800/1000-m run)—are defined and measured. |
| Data sources/ measurement | 8* | For each variable of interest, give sources of data and details of methods of assessment (measurement). Describe comparability of assessment methods if there is more than one group | 4 | Tools like the Perceived Physical Literacy Instrument (PPLI-SC) and Physical Activity Questionnaire for Adolescents (PAQ-A) are used, with detailed measurement methods provided. |
| Bias | 9 | Describe any efforts to address potential sources of bias | 5 | Steps to reduce bias are outlined, such as standardizing recruitment and data collection processes |
| Study size | 10 | Explain how the study size was arrived at | 5 | The study size of 115 participants was determined based on power analysis, which ensured sufficient statistical power to detect the expected effects. Additionally, the expected response rate was taken into account, and the sample size was adjusted accordingly to account for potential non-responses. |

Continued on next page

| Quantitative variables | 11 | Explain how quantitative variables were handled in the analyses. If applicable, describe which groupings were chosen and why | 5 | Quantitative variables (e.g., BMI, fitness indicators) are handled using statistical methods such as regression analysis. |
| --- | --- | --- | --- | --- |
| Statistical methods | 12 | (*a*) Describe all statistical methods, including those used to control for confounding | 5 | Statistical analysis was performed using SPSS Statistics 26.0:  **Descriptive statistics** for demographic and fitness data (mean ± SD, frequencies).  **Pearson's correlation** to assess relationships between PL, PALs, and fitness indicators.  **Hierarchical regression** to examine PL's effect on PALs and its mediation role in fitness outcomes.  **Confounders (age, gender)** were controlled in regression models. |
|  |  | (*b*) Describe any methods used to examine subgroups and interactions | 5 | **Subgroup analysis** was performed by gender using independent-samples t-tests to examine differences in PL, PALs, and fitness. Interaction terms were tested in regression models to explore gender differences in the relationships between PL and fitness. |
|  |  | (*c*) Explain how missing data were addressed | 5 | **Listwise deletion** was used to handle missing data, excluding participants with missing values on any key variables. This approach was selected due to minimal missing data and minimal impact on the results. |
|  |  | (*d*) *Cohort study*—If applicable, explain how loss to follow-up was addressed  *Case-control study*—If applicable, explain how matching of cases and controls was addressed  *Cross-sectional study*—If applicable, describe analytical methods taking account of sampling strategy | 5 | **Cluster sampling** was used to select classes, with all students in selected classes invited. No additional adjustments for sampling strategy were needed for analysis, as all participants within clusters were included. |
|  |  | (*e*) Describe any sensitivity analyses |  | A **sensitivity analysis** using bootstrapping (5000 resamples) was conducted in the PROCESS macro for SPSS to confirm the robustness of the mediation results, which remained consistent across multiple analyses. |
| Results | | | | |
| Participants | 13* | (a) Report numbers of individuals at each stage of study—eg numbers potentially eligible, examined for eligibility, confirmed eligible, included in the study, completing follow-up, and analysed | 6 | **Potentially eligible:** 200 first-year students from randomly selected classes.  **Examined for eligibility:** 115 students met the eligibility criteria.  **Confirmed eligible:** 115 students confirmed eligibility.  **Included in the study:** 115 students participated.  **Completing follow-up:** Not applicable (cross-sectional study).  Analyzed: 115 students' data were included in the analysis. |
|  |  | (b) Give reasons for non-participation at each stage | 6 | There were no non-participation issues. All eligible students (115) agreed to participate in the study. |
|  |  | (c) Consider use of a flow diagram | 6 | A flow diagram was not included in the current study, but it could be added in future versions for clarity, showing recruitment, eligibility screening, and participation stages. |
| Descriptive data | 14* | (a) Give characteristics of study participants (eg demographic, clinical, social) and information on exposures and potential confounders | 6 | **Demographic:** 115 first-year university students (79 males, 36 females), aged 18-22.  **Exposures:** Physical literacy (PL) and physical activity levels (PALs), measured using PPLI-SC and PAQ-A, respectively.  **Potential confounders:** Age and gender were included as potential confounders in the analyses.· |
|  |  | (b) Indicate number of participants with missing data for each variable of interest | 6 | There were no missing data for any of the key variables (PL, PALs, or physical fitness indicators) in the study. |
|  |  | (c) *Cohort study*—Summarise follow-up time (eg, average and total amount) | 6 | This is a **cross-sectional study**, so there was no follow-up period. Data were collected at a single time point between October 2024 and January 2025. |
| Outcome data | 15* | *Cohort study*—Report numbers of outcome events or summary measures over time | *7* | This is a **cross-sectional study**, so there are no outcome events or summary measures reported over time. |
|  |  | *Case-control study—*Report numbers in each exposure category, or summary measures of exposure | *7* | This is a cross-sectional study, so exposure categories are not reported in this format. |
|  |  | *Cross-sectional study—*Report numbers of outcome events or summary measures | *7* | Outcome events or summary measures for physical fitness indicators are as follows:  BMI: Mean = 21.6 ± 3.62 kg/m^2^  50 m sprint: Mean =7.67 ± 0.51s  Sit-and-reach: Mean = 12.10 ± 4.71 cm  Endurance run (800m/1000m): Mean = 226.25 ± 24.75 s. |
| Main results | 16 | (*a*) Give unadjusted estimates and, if applicable, confounder-adjusted estimates and their precision (eg, 95% confidence interval). Make clear which confounders were adjusted for and why they were included | 8 | **Unadjusted estimates:** The correlation between PL and PALs was r = 0.55 (p < 0.01).  **Confounder-adjusted estimates:** After adjusting for age and gender, the relationship between PL and PALs remained significant with r = 0.52 (95% CI: 0.48 to 0.57). Age and gender were adjusted for as they are potential confounders that may influence both PL and PALs. |
|  |  | (*b*) Report category boundaries when continuous variables were categorized | 8 | BMI:  Underweight: BMI < 18.5  Normal weight: 18.5 ≤ BMI < 24.9  Overweight: BMI ≥ 25 |
|  |  | (*c*) If relevant, consider translating estimates of relative risk into absolute risk for a meaningful time period | 8 | As this is a cross-sectional study, relative risk estimates were not applicable. The study focused on correlation and mediation rather than predicting absolute risks over time. |

Continued on next page

| Other analyses | 17 | Report other analyses done—eg analyses of subgroups and interactions, and sensitivity analyses | 8-9 | **Subgroup analyses:** Gender-based subgroup analysis was conducted to assess differences in the relationships between PL and PALs.  **Interaction analyses:** Interaction terms for gender and PL were tested in regression models, showing no significant gender differences.  **Sensitivity analysis:** Bootstrapping (5000 resamples) was performed in mediation analysis, confirming the stability of the results. |
| --- | --- | --- | --- | --- |
| Discussion | | | | |
| Key results | 18 | Summarise key results with reference to study objectives | 9 | The study found that physical literacy (PL) was positively correlated with physical activity levels (PALs) (r = 0.55, p < 0.01). PALs fully mediated the relationship between PL and physical fitness outcomes, including explosive power, flexibility, and cardiorespiratory endurance. No significant direct relationship was found between PL and BMI. The study objectives were met by demonstrating that PALs play a key role in translating PL into improved fitness outcomes. |
| Limitations | 19 | Discuss limitations of the study, taking into account sources of potential bias or imprecision. Discuss both direction and magnitude of any potential bias | 9 | The cross-sectional design limits causal conclusions. Sampling bias may occur as participants were from a single university, reducing generalizability. Self-reported data from the PAQ-A may introduce measurement bias. The sample size (n = 115) may limit precision, particularly in subgroup analyses. However, the biases' impact is likely minimal due to standardized methods and robust analysis. |
| Interpretation | 20 | Give a cautious overall interpretation of results considering objectives, limitations, multiplicity of analyses, results from similar studies, and other relevant evidence | 9 | The study suggests that physical literacy (PL) is positively related to physical activity levels (PALs), which, in turn, mediates the relationship between PL and physical fitness outcomes. However, due to the cross-sectional design, causality cannot be established. The sample size and potential self-report bias limit the precision of some results. Similar studies support the mediating role of PALs, but further research with larger samples and longitudinal designs is needed to confirm these findings and explore causal pathways. |
| Generalisability | 21 | Discuss the generalisability (external validity) of the study results | 10 | The study's findings may have limited generalizability as participants were drawn from a single university, potentially not representing broader populations. The sample size (n = 115) and the specific demographic (university freshmen) may also limit the applicability to other age groups or settings. Further research with diverse and larger samples is needed to confirm whether these results apply to different populations. |
| Other information | |  | | |
| Funding | 22 | Give the source of funding and the role of the funders for the present study and, if applicable, for the original study on which the present article is based | 10 | The study was funded by the Major Research Project in Philosophy and Social Sciences of Jiangsu Province Universities, China (grant number 2023SJZD142)  Fundamental Research Funds for the Central Universities (grant number SKCX2023016)  Additionally, the funders had no role in study design, data collection and analysis, decision to publish, or preparation of the manuscript . |

*Give information separately for cases and controls in case-control studies and, if applicable, for exposed and unexposed groups in cohort and cross-sectional studies.

**Note:** An Explanation and Elaboration article discusses each checklist item and gives methodological background and published examples of transparent reporting. The STROBE checklist is best used in conjunction with this article (freely available on the Web sites of PLoS Medicine at http://www.plosmedicine.org/, Annals of Internal Medicine at http://www.annals.org/, and Epidemiology at http://www.epidem.com/). Information on the STROBE Initiative is available at www.strobe-statement.org.
