# Supplementary material for: Exploring the mediating role of physical activity levels in the relationship between physical literacy and physical fitness in Chinese university students
Source: PeerJ. 2026 Feb 3;14:e20689. doi: 10.7717/peerj.20689 (PMC12880097; doi:10.7717/peerj.20689)
Supplement: Supplemental Information 3 [file peerj-14-20689-s003.docx]

**Codebook for Dataset Variables**

# English Version

Sex
- 1 = Male
- 2 = Female

Age
- 17 = 17 years old
- 18 = 18 years old
- 19 = 19 years old

**H (Height, cm):** Continuous variable, measured in centimeters.
**W (Weight, kg):** Continuous variable, measured in kilograms.
**BMI (Body Mass Index):** Continuous variable, calculated as weight/height² (kg/m²).
**EP (Explosive Power, 50-m sprint):** Continuous variable, measured in seconds.
**CE (Cardiorespiratory Endurance, 800/1000-m run):** Continuous variable, measured in seconds.
**F (Flexibility, sit-and-reach test):** Continuous variable, measured in centimeters.
**PALs (Physical Activity Levels):** Continuous variable, mean score from PAQ-A questionnaire (1–5).
**PL (Physical Literacy):** Continuous variable, total score from PPLI-SC (higher = better).
